# Transforming Health and Reducing Perinatal Anxiety Through Virtual Engagement: Protocol for a Randomized Controlled Trial

**DOI:** 10.2196/70627

**Published:** 2025-05-30

**Authors:** Carolyn Ponting, Rebecca J Baer, Kacie Blackman, Bridgette Blebu, Jennifer N Felder, Scott Oltman, Karen M Tabb, Laura Jelliffe Pawlowski

**Affiliations:** 1 Department of Psychiatry and Behavioral Sciences University of California San Francisco San Francisco, CA United States; 2 Department of Obstetrics, Gynecology, and Reproductive Health Sciences University of California San Francisco San Francisco, CA United States; 3 Department of Pediatrics University of California San Diego San Diego, CA United States; 4 California Preterm Birth Initiative University of California San Francisco San Francisco, CA United States; 5 Department of Health Sciences College of Health and Human Development California State University, Northridge Northridge, CA United States; 6 Lundquist Institute for Biomedical Innovation Harbor–UCLA Medical Center Torrance, CA United States; 7 Osher Center for Integrative Health University of California San Francisco San Francisco, CA United States; 8 Department of Epidemiology and Biostatistics University of California San Francisco San Francisco, CA United States; 9 Institute for Global Health Sciences University of California San Francisco San Francisco, CA United States; 10 School of Social Work University of Illinois Urbana-Champaign Urbana, IL United States; 11 Rory Meyers College of Nursing New York University New York, NY United States

**Keywords:** cognitive behavioral therapy, telemedicine, perinatal care, anxiety, ethnic and racial minorities

## Abstract

**Background:**

Prenatal anxiety affects between 20% and 30% of pregnant people and is associated with adverse prenatal health conditions, birth outcomes, and postpartum mental health challenges. Individuals from racial and ethnic minority groups, sexual and gender minority groups, and those with low income are all at heightened risk for prenatal anxiety due to disproportionate exposure to adverse social determinants of health. Digital cognitive behavioral therapy (dCBT) has been shown to reliably reduce anxiety in mostly White and middle- to higher-income samples, but its efficacy in low-income and marginalized pregnant people is understudied.

**Objective:**

We propose a randomized controlled trial of a dCBT (Daylight app, Big Health, Ltd) in a sample of low-income pregnant people oversampled for racial, ethnic, sexual, and gender minority identity.

**Methods:**

Participants (N=132) will be randomized to the intervention or waitlist control group using a 1:1 allocation ratio. The intervention will be a self-guided application that uses an online therapist to teach and encourage the practice of 4 key cognitive behavioral therapy skills (eg, identifying catastrophic thinking and increasing physical relaxation) that can reduce anxiety. The primary outcome will be generalized anxiety symptoms; secondary outcomes will include depressive symptoms, stress, pregnancy-specific anxiety, and insomnia symptoms. Focus groups with a subset of participants will provide qualitative data about the acceptability of dCBT.

**Results:**

Recruitment began in June 2024. Data will be analyzed using linear mixed models, which will be fit with treatment condition (dCBT and waitlist control group) as the between-group factor, time (baseline, 3, 6, and 10 weeks post randomization) as a within-group factor, and a group-by-time interaction. Linear mixed models produce unbiased parameter estimates in situations where there are different numbers of observations per record and will accommodate intent-to-treat and sensitivity analyses.

**Conclusions:**

This clinical trial will evaluate the efficacy and acceptability of a self-guided dCBT for prenatal anxiety among low-income and marginalized pregnant people, a group that continues to experience substantial barriers to accessing in-person evidence-based psychotherapy.

**Trial Registration:**

ClinicalTrials.gov NCT06404450; https://clinicaltrials.gov/study/NCT06404450

**International Registered Report Identifier (IRRID):**

DERR1-10.2196/70627

## Introduction

Anxiety is common and consequential during pregnancy; globally, about 15% of pregnant people are diagnosed with an anxiety disorder, and 18%-25% of pregnant people self-report symptoms that surpass a clinical cutoff using a validated screener [1]. Symptoms of clinical anxiety include experiencing excessive worry, difficulty controlling worries, restlessness, irritability, and sometimes, physical symptoms, such as heart palpitations and dizziness [2]. Anxiety symptoms can be exacerbated by experiences common during pregnancy, which include poor sleep and managing uncertainties related to labor and delivery [3]. The COVID-19 pandemic had profound impacts on the prevalence rates of prenatal anxiety. Meta-analytic data showed that about 31% of pregnant people reported elevated anxiety symptoms during the COVID-19 pandemic compared to 22.4% before the pandemic, representing a 35% increase in prevalence [2,3]. Notably, increase in the rates of elevated prenatal anxiety and pandemic-related symptoms did not impact pregnant people equally. Elevations in symptoms of anxiety and comorbid symptoms like depression have persisted at higher rates in marginalized pregnant people, including those who are categorized as Black, Latinx, Indigenous, Asian and Pacific Islander, and lesbian, gay, bisexual, transgender, and queer (LGBTQ+), and in those with low income [4,5].

Elevated anxiety symptoms during pregnancy contribute to the risk of a host of adverse prenatal health conditions, birth outcomes, and postpartum mental health problems. For example, prenatal anxiety is prospectively associated with gestational hypertension, intrauterine growth restriction, and having a preterm birth [6,7]. Biological mechanisms, like dysregulation in the hypothalamic-pituitary axis leading to premature accelerations in placental corticotropin-releasing hormones and cortisol, and behavioral mechanisms, such as substance use and poor sleep help explain the relationship between prenatal anxiety and adverse birth outcomes [8]. Elevated anxiety is also associated with psychiatric comorbidities, including depression, insomnia, and suicidal ideation [9-11], which are more common in marginalized pregnant people and those with low income [4,5,12,13].

In addition to anxiety disorders and elevations in general anxiety symptoms, pregnant people also experience pregnancy-specific anxiety*,* an affective state of persistent worry surrounding a pregnant person’s prenatal health, delivery, the well-being of the baby, and future parenting [14]. Pregnancy-specific anxiety is estimated to affect between 14% and 19% of pregnant people [14,15] and appears to be uniquely predictive of adverse birth outcomes like preterm birth. For example, pregnancy-specific anxiety is related to smaller gestational age and lower birth weight after accounting for generalized anxiety disorders [16]. Further, it is estimated that women with high levels of pregnancy-specific anxiety are 1.7-2.5 times more likely to experience a spontaneous preterm birth than women without pregnancy-specific anxiety [17]. Unsurprisingly, pregnant people reported high rates of pregnancy-specific anxiety during the COVID-19 pandemic [18]—a time when practices in labor and delivery units were in flux, and people worried about what a COVID-19 infection might mean for their developing fetus. With respect to sociodemographic risk, greater pregnancy-specific anxiety has been linked with lower income, non-White race, and Latina ethnicity [19-21]. These patterns suggest that rising rates of clinical and pregnancy-specific anxiety may be contributing to worsening racial and ethnic, LGBTQ+, and socioeconomic disparities in adverse pregnancy outcomes and postpartum mental health. Given the high need and lack of representation of racial and ethnic minoritized, marginalized, and low-income people in randomized trials for perinatal anxiety to date, testing the efficacy and acceptability of accessible interventions that address clinical anxiety among these groups is warranted [22,23].

Therapist-delivered cognitive behavioral therapy (CBT) has been shown to be effective in treating anxiety among White nonpregnant [24] and pregnant populations [25]. However, the effects of CBT on pregnancy-specific anxiety are largely unknown, as most studies testing interventions for anxiety in pregnancy do not measure this subtype of anxiety [26]. Notwithstanding, cognitive behavioral interventions can improve biological and behavioral variables that link pregnancy-specific anxiety to adverse birth and postpartum outcomes. For example, cognitive behavioral interventions can reduce cortisol [27] and improve sleep [28] in pregnant people.

Despite the promise of CBT for elevated anxiety symptoms experienced during pregnancy, racial and ethnic minoritized, marginalized, and low-income pregnant people often encounter several barriers to accessing evidence-based treatment (eg, long waitlists, childcare issues, and challenges with transportation) [29]. Recent innovations have focused on addressing barriers to CBT by adapting it for automated, digital delivery. Digital CBT (dCBT) is efficacious for treating clinical anxiety in mostly White, higher socioeconomic status, pregnant [30], and nonpregnant populations [31]. While marginalized pregnant people report high satisfaction with digital therapy, very few dCBT trials have enrolled meaningful numbers of racial and ethnic minoritized or low-income pregnant people [32,33]. This study addresses the critical need to evaluate whether dCBT can be used to address clinical anxiety among racial and ethnic minoritized, marginalized, and low-income pregnant people, subgroups disproportionately impacted by anxiety during pregnancy.

Building on a previous efficacy trial of the same dCBT in nonpregnant adult women that evidenced large improvements in anxiety symptoms, and high levels of engagement and safety [31], the Transforming Health and Reducing Perinatal Anxiety through Virtual Engagement (THRIVE) study tests whether the dCBT Daylight (Big Health), is an efficacious and acceptable treatment during pregnancy among racial and ethnic minoritized, marginalized, and low-income people. The hypothesis of this trial is that participants randomized to Daylight will demonstrate significantly greater reductions in anxiety symptoms compared to a waitlist control group. We also expect that participants randomized to Daylight will report reductions in associated mental health symptoms, including depression, stress, pregnancy-specific anxiety, and insomnia.

## Methods

### Objectives

Study objectives are to (1) measure the impact of dCBT (Daylight) on anxiety symptoms in racial and ethnic minoritized, marginalized, and low-income pregnant people with elevated anxiety; (2) assess the acceptability of dCBT for addressing elevated anxiety and related symptoms among pregnant participants; and (3) identify barriers and facilitators to the adoption of dCBT for use by racial and ethnic minoritized, marginalized, and low-income pregnant people enrolled in public insurance (MediCal).

### Trial Design

#### Overview

This is a phase 1 randomized controlled trial (RCT) that tests the efficacy of dCBT (Daylight) for treating anxiety during pregnancy using a parallel group design. Study participants will be in the study from enrollment at 8-27 weeks gestation until 6-8 weeks after their expected date of delivery. All participants complete assessments of self-reported symptoms of generalized anxiety, depression, stress, pregnancy-specific anxiety, and insomnia at baseline, 3, 6, and 10 weeks following randomization, and at a follow-up time point between 6 and 8 weeks post partum. The study also includes focus groups with a subset of RCT participants following intervention receipt to evaluate the acceptability of dCBT.

#### Randomization and Blinding

As outlined in the study schema (Table 1), after completion of the study consent and enrollment survey, participants will receive an email informing them if they have been randomized to receive complimentary access to the Daylight app immediately (ie, intervention condition), or to receive access in 10 weeks (ie, waitlist control condition). If randomized to receive access immediately, they will receive instructions on how to download the app and start using it. If randomized to receive access to the Daylight app in 10 weeks, they will be sent instructions for downloading and starting to use the Daylight app 10 weeks later.

**Table 1:** Modules included in the Daylight digital cognitive behavioral therapy app.

Intervention module	Brief description	CBT^a^ principle	Learning goals
Worry scheduling and worry time	Scheduling a specific time and pace to worry (Worry Scheduling), and then cycling through worrisome thoughts (Worry Time).	Stimulus control	Learn to associate worry with a designated time and space. Learn to delay worry until a designated time. Learn to tolerate worry without avoidance.
Tense and release	Tensing and releasing muscles with relaxation cues.	Applied relaxation	Increase awareness of tension. Increase awareness of the difference between tensed and relaxed muscles. Learn to reduce muscle tension.
Thought challenger	Identifying catastrophic or unhelpful thoughts and using challenge questions to develop new perspectives on these thoughts.	Cognitive restructuring (decatastrophizing)	Increase awareness of catastrophic and unhelpful thoughts. Learn to challenge catastrophic thoughts. Increase cognitive flexibility.
Worry exposure	Imagining the “worst case scenario” or feared outcome of a specific worry.	Imaginal exposure	Learn to tolerate emotions associated with feared outcomes. Facilitate cognitive reappraisal of feared outcomes. Facilitate habituation to fear response (ie, fear extinction).

^a^CBT: cognitive behavioral therapy.

This study is designed to enroll marginalized (Latinx; Black, Indigenous, and people of color; or LGBTQ+ individuals) individuals at a ratio of 2:1. To ensure that randomization is in keeping with our intention of equal representation across groups, we will use a 1:1 allocation ratio with blocked randomization of marginalized people and not. The randomization sequence will be generated by the study biostatistician. The randomization sequence and block sizes will be concealed from all other study team members and participants. Study team members will not be able to influence randomization. Once an eligible participant completes their consent and enrollment survey, unmasked study staff will reveal the allocation assignment and notify participants of their assigned condition. The principal investigator and any staff member involved in data analysis will be blinded to condition assignment. Although we cannot blind participants from condition assignment, allocation will occur after the participant has enrolled to avoid selection bias.

### Participants

#### Enrollment and Eligibility

This is a remote RCT overseen at the University of California, San Francisco, and pregnant people residing in California are eligible to take part. In addition to California residence, participants must meet the following eligibility criteria: (1) enrollment in MediCal, a California health insurance plan for low-income people; (2) currently between 8 and 27 weeks pregnant; (3) age 18 years or older; (4) able to speak in English; (5) ninth-grade levels of education or greater; (6) daily access to a web-enabled computer, smartphone or tablet; and (7) elevated anxiety symptoms as measured by a score of 10 or greater on the Generalized Anxiety Disorder- 7 Scale (GAD-7; [34]). Consistent with the original Daylight efficacy trial [31], participants who have received CBT treatment for anxiety in the last 12 months, those with a new or changed dose of prescription medication for anxiety, depression, or insomnia in the last 4 weeks, and those with a self-reported diagnosis of schizophrenia, psychosis, bipolar disorder, seizure disorder, and substance use disorder will be excluded. A recent head trauma or brain damage, or a serious physical health concern necessitating surgery or hospitalization in the last 6 months will also lead to exclusion from the study.

#### Sample Size

In total, 110 pregnant people with elevated anxiety symptoms will be randomized to one of 2 study arms. Based on a previous study wherein the response to the Daylight tool (by the GAD-7; [34]) at 10 weeks post randomization was normally distributed with a mean of 12.15 (SD 4.73) in the waitlist control, and a mean of 7.90 (SD 4.85) in the dCBT group [31], we expect that a sample of 55 experimental and 55 control participants should detect a true difference in the mean symptoms of experimental and control participants of –2.696 or 2.696 with 80% power. An additional 22 participants will be recruited (ie, a total of 132 enrolled) to account for attrition rates of 17%, the average rate of attrition of marginalized pregnant people enrolled in psychological intervention studies [35]. While we are powered to detect intervention effects at 2 posttreatment time points (6 weeks and 10 weeks following randomization), we do not have pilot data to estimate power at our postpartum time point. Estimates of an effect size in this perinatal pilot are challenging to calculate, as gestational age may vary widely at enrollment (8-27 weeks) resulting in a postpartum assessment that will be up to 36 weeks post treatment for those enrolling at 8 weeks and as early as 15 weeks post treatment for those enrolling at 27 weeks. Thus, treatment effects at the 7-week postpartum time point are exploratory, and we will rely on effect sizes to describe effects as opposed to *P* values.

#### Recruitment

Recruitment methods include collaborations with the Postpartum Support International (PSI) and with the University of California, San Francisco (UCSF) Clinical & Translational Science Institute (CTSI). These partners will disseminate study information via emails to participants and partners in their programs, with affiliate organizations and community partners using a study-specific flyer, and by social media advertising.

### Intervention and Comparator

#### Daylight

Participants who are randomly assigned to receive dCBT will be provided with complimentary access to Daylight [36]. The program uses an online therapist to guide individuals through interactive exercises and animations to facilitate the learning and implementation of 4 key CBT techniques. The four CBT techniques are cognitive (eg, identifying catastrophic thinking) and behavioral (eg, increasing physical relaxation and reducing avoidance) skills that can reduce anxiety with repeated use. At present, the Daylight app is only available in English. See Table 1 for a more detailed description of each intervention module.

Daylight is designed to be self-paced, with the app encouraging users to practice techniques both within the app and in real-life situations on a daily basis. Upon signing up, users automatically receive reminders and motivational messages via email and can choose to opt in for additional communication through text messages and push notifications to encourage daily use and intervention adherence, a prominent challenge in digital interventions [37]. Each of the 4 modules takes between 10 and 20 minutes to complete. In addition, shorter 5-minute exercises guide users through various skills (eg, progressive muscle relaxation) and are designed for repeated use over the intervention period. Weekly in-app assessments of anxiety, depressive symptoms, and sleep are also used, allowing for personalized feedback based on users’ self-reported symptoms. For example, if a user reports difficulties with sleep, they may receive prompts to engage in relaxation techniques at bedtime. Daylight provides objective metrics for engagement, including the number of modules completed, the frequency with which the app is accessed, and the time spent to complete modules. All participants in THRIVE will receive access to Daylight for 12 months after delivery (ie, until their baby is a year old).

#### Waitlist Control

The waitlist control group participants will get complimentary access to the Daylight program after completing the 10-week follow-up survey. A waitlist control condition was chosen to ensure that the entire low-income sample, a group with documented health disparities during the perinatal period [38], would have access to the intervention. Allowing access to the intervention 10 weeks following randomization allows for access to treatment in pregnancy and reduces the risk of participant dropout, which tends to increase the longer participants are asked to wait to receive treatment [39]. All participants can also continue using other pharmacological or nonpharmacological interventions (excluding CBT) for anxiety or other symptoms.

### Study Procedures and Timeline

#### Initial Screening and Orientation

Before enrolling, candidate participants will complete a 3-part screening and orientation process. First, participants will complete a screening consent form, followed by a screening questionnaire that will assess all inclusion and exclusion criteria. Second, candidate participants will verify key information submitted on the screening questionnaire via email (eg, expected due date and prenatal care provider) to minimize the risk of fraudulent screeners. Finally, candidate participants will attend a live online group orientation session where a staff member will provide an overview of the study and provide participants the opportunity to ask questions. This multistep process has maximized study engagement and retention in previous longitudinal RCTs [40].

#### Assessment Collection and Study Timeline

All participants, regardless of randomization grouping, will receive surveys that take approximately 20 minutes to complete at enrollment and at 3, 6, and 10 weeks after randomization that asks about health and well-being as well as about current experiences of anxiety, stress, depression, and sleep. As described in Table 2, demographic information collected at screening and enrollment includes information about race and ethnicity, insurance, housing stability, and food security. Pregnancy history and data about any previous preterm births, cesarean sections, or preeclampsia are collected only at enrollment. Information about pregnancy health and the diagnosis of conditions like gestational hypertension and gestational diabetes is collected across all time periods. Information about medical care and social support is also consistently collected across time periods.

**Table 2:** Overview of surveys and schedule of data collection for the Transforming Health and Reducing Perinatal Anxiety through Virtual Engagement (THRIVE) phase I randomized clinical trial

Instrumentation		Time point					
Measure	Validated for use in pregnancy	Screening	Enrollment	3 weeks+/- 1 week	6 weeks+/- 1 week	10 weeks+/- 1 week	7 weeks after delivery+/- 1 week
Demographics	—^a^	✓	✓				✓
Pregnancy outcome and history	—		✓				
Pregnancy health	—	✓	✓	✓	✓	✓	✓
Care and social support	—		✓	✓	✓	✓	✓
Labor and delivery experience	—						✓
Infant health and wellbeing	—						✓
GAD-7^b^	Yes	✓		✓	✓	✓	✓
PSS-4^c^	Yes		✓	✓	✓	✓	✓
PRAS^d^	Yes		✓	✓	✓	✓	
ISI^e^	Yes		✓	✓	✓	✓	✓
PHQ-9^f^	Yes		✓	✓	✓	✓	✓

^a^Not applicable.

^b^GAD-7: Generalized Anxiety Disorder 7 Scale.

^c^PSS-4: Perceived Stress Scale 4.

^d^PRAS: Pregnancy Related Anxiety Scale.

^e^ISI: Insomnia Severity Index.

^f^PHQ-9: Patient Health Questionnaire-9.

**Figure 1:**
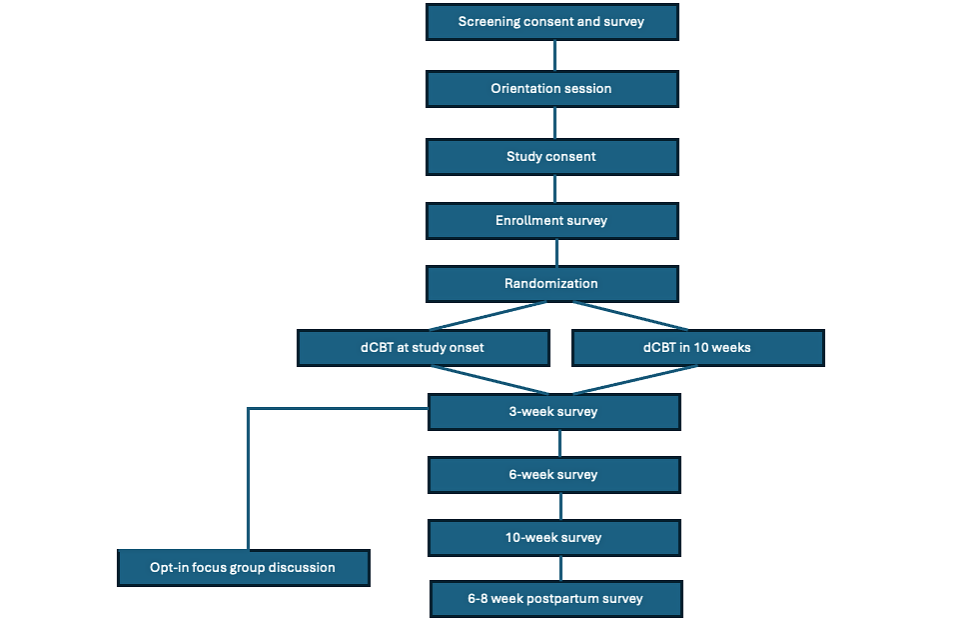
Study schema for randomized controlled trial and optional focus group discussion. dCBT: digital cognitive behavioral therapy.

The final survey at 6-8 weeks after the expected date of delivery will ask participants to report on symptoms of anxiety, stress, depression, and insomnia as well as their experience of labor and delivery and the health of their baby. All surveys will alert participants to missing items to reduce missing data resulting from participant error. Participants will be contacted via email, phone, or text message, according to participant preference, with reminders to complete their surveys. Following the completion of each of the 4 surveys, participants will receive a US $50 electronic gift card sent via email. See Figure 1 for a schematic representation of the RCT and the optional focus group discussions.

### Ethical Considerations

#### Overview

This study has been approved by the Western Copernicus Group (WGC) institutional review board (IRB; approval number 1364733) and registered in the Clinical Trials Registry of the National Institutes of Health National Library of Medicine (protocol number NCT06404450). The clinical protocol for THRIVE (version 1.1) was IRB-approved and has not been changed since November 14, 2023. This study protocol was guided by the SPIRIT (Standard Protocol Items: Recommendations for Interventional Trials), 2013 statement [41]; the SPIRIT checklist is available in Multimedia Appendix 1. All roles and responsibilities of study personnel and study funders are described in Multimedia Appendix 2.

#### Informed Consent and Privacy Considerations and Data Monitoring

Informed consent will be obtained from pregnant women who qualify for the study after initial screening and following participation in a scheduled orientation session. Participants will complete consent forms (see Multimedia Appendix 3) online with the capture of electronic signatures. All potential participants will be offered the opportunity to complete consent forms with the help of study staff. Information in the consent form is also reviewed at the orientation session attended by eligible participants.

Personal identifying information (eg, first and last name, date of birth, phone number, email address, and postal address) will be collected at the time of enrollment and stored separately from all other study data with linkage only by a study ID, which is only available to a small set of study personnel. Participants will be instructed to complete all research activities in a private setting. In addition, participants will be assigned unique, coded, confidential identifiers that will be used to label all data forms, data entries, and questionnaires. The key linking participant identity to their unique coded identifier will be stored on REDCap (Research Electronic Data Capture; Vanderbilt University). REDCap is a secure, HIPAA (Health Insurance Portability and Accountability Act)-compliant web-based software platform designed to support data capture for research studies, providing (1) an intuitive interface for validated data capture, (2) audit trails for tracking data manipulation, and (3) automated export procedures for seamless data downloads to common statistical packages [42]. Access to all data will be limited to the principal investigator and trained, authorized study staff. All data (recordings, transcripts, surveys, coded data) for the study are stored on encrypted servers kept behind UCSF firewalls, where access is by remote access with dual authenticated password protection. No study data is kept or accessible on local devices.

## Results

Grant funding was received in July 2023. As of March 2025, a total of 24 participants were confirmed eligible and randomized. We expect that data analysis and results will be available in May 2026.

### Primary Outcome: Anxiety Symptoms

Anxiety is self-reported using the GAD-7 [34]. The GAD-7 measures the frequency of generalized anxiety symptoms in the previous 2 weeks. Items are scored on a 4-point Likert scale (0=not at all to 3=nearly every day) and summed for a possible total score of 0-21, where higher scores indicate greater elevations in clinically significant anxiety. Cutoff points for the GAD-7 indicate that scores totaling 5-9 correspond to minimal anxiety; 10-14, to moderate anxiety; and 15-21, to severe anxiety. The GAD-7 shows strong psychometric properties, including sensitivity, specificity, and internal consistency in nonpregnant [34,43] and pregnant adults [44].

### Secondary Outcomes

#### Depressive Symptoms

Depression is self-reported using the 9-item Patient Health Questionnaire-9 (PHQ-9 [45]). The PHQ-9 measures the frequency of depressive symptoms over the past 2 weeks. Items are scored on a 4-point Likert scale (0=not at all to 3=nearly every day) and summed for a possible total score of 0-27, with higher scores indicating greater depression severity. Cutoff points for the PHQ-9 indicate that scores totaling 5-9 correspond to minimal depression; 10-14, to moderate depression; 15-19, to moderately severe depression; and 20-27, to severe depression. The PHQ-9 is the most widely used depression screening measure globally and shows convergent and criterion validity as well as good sensitivity and specificity among pregnant and postpartum samples [46].

#### Perceived Stress

Perceived stress is self-reported using the 4-item Perceived Stress Scale (PSS-4) [47]). The PSS measures the frequency of stressful thoughts and feelings over the past month. Items are scored on a 5-point Likert scale (0=never, 4=very often) and summed for a possible total score of 0-16, with higher scores indicating higher perceived stress. The PSS shows internal consistency and construct validity among several adult samples [48] as well as convergent and concurrent validity and internal consistency in pregnant people [49].

#### Pregnancy-Specific Anxiety

Pregnancy-specific anxiety is measured using the 10-item Pregnancy-Related Anxiety Scale (PRAS-10) [50]). The PRAS measures the frequency and severity of pregnant people’s present concern, worry, or fear about various aspects of their pregnancy and delivery*.* Items are scored on a 4-point Likert scale (1=never, 4=almost all the time), with a possible total score of 4-40, where higher scores indicate greater pregnancy-specific anxiety. The PRAS shows good reliability and internal consistency across diverse samples of pregnant people [21,50,51].

#### Insomnia Symptoms

Insomnia is self-reported using the 7-item Insomnia Severity Index (ISI-7 [52]). The ISI measures the nature, severity, and impact of insomnia symptoms over the last month. Items are scored on a 5-point Likert scale (0=no problem, 4=very severe problem), with a possible total score of 0-28, with higher scores indicating greater insomnia severity. The ISI shows excellent internal consistency and reliably distinguishes between clinical and nonclinical sleep problems [53]. It is also considered reliable, and structurally invariable in pregnant people [54].

#### Safety and Adverse Events

Per the UCSF Human Research Protection Program, an adverse event is defined as any untoward or unfavorable medical occurrence in a human participant, including any abnormal laboratory finding, symptom, or disease, temporally associated with the individual’s participation in the research, whether or not considered related to the individual’s participation in the research. All adverse events will have their relationship to study procedures, including the intervention, assessed by an appropriately trained clinician based on temporal relationship and clinical judgment. Adverse event reporting will be done in keeping with the WCG IRB Policy HRP-071. Specifically, adverse events will be reported within 5 calendar days of principal investigator awareness when the adverse event requires a change to the protocol or consent document. For additional information regarding documentation and definition of adverse events in the THRIVE trial, see Multimedia Appendix 4.

#### Analyses

To examine the efficacy of dCBT (Daylight) on generalized anxiety symptoms (primary outcome) and symptoms of depression, stress, pregnancy-specific anxiety, and insomnia (secondary outcomes) from baseline to 10 weeks post randomization, we will use LMMs. These LMMs will be fit with treatment condition (dCBT and waitlist control group) as the between-group factor, time (baseline, 3, 6, and 10 weeks post randomization) as a within-group factor, and a group-by-time interaction. LMMs maximize data use because they produce unbiased parameter estimates in situations where there are different numbers of observations per record, so long as data are missing at random. A model-based approach will be used to assess the potential impact of data missing not at random on our findings. Pattern mixture models will be used to identify missingness patterns (eg, differential attrition based on randomization group) and to stratify analysis by these patterns as needed [55]. All primary and secondary analyses will be conducted on all randomized participants using an intention-to-treat approach. Sensitivity analyses will also be conducted to test the effects of Daylight among participants who (1) download the app, (2) complete at least 2 modules, and (3) complete all 4 modules, mirroring planned analyses from the original Daylight efficacy trial [56]. Finally, dependent on sample size, exploratory moderation analyses will be conducted to test for differential intervention efficacy among subgroups of racial, ethnic, or sexual and gender minoritize. No interim analyses are planned.

Regarding the assessment of dCBT acceptability, focus groups will be transcribed for qualitative analysis. In line with implementation science frameworks, we will use rapid qualitative techniques for thematic network analysis [57] using NVivo (version 11.4.2; Lumivero) software. Following the identification of codes, the 4 raters will meet to discuss differences and similarities amongst codes to ensure consistency. Codes will be refined and discussed until consensus amongst raters is reached.

#### Community Engagement and Dissemination

Study investigators engaged with community members who were part of a previous study focused on the biopsychosocial factors related to health in pregnancy [58] to identify treatments for anxiety as a community priority. Investigators will continue to engage with community members on outreach activities, including presentations to stakeholder groups. Investigators will also work with community partners on the next steps for disseminating dCBT to low-income communities if data from the trial support its efficacy and acceptability. Study results will be reported in one or more medical journals, presented to the funder, and interested stakeholders and community groups. Once published, we will also share a plain language summary of the results with study participants.

## Discussion

### Principal Findings

This randomized controlled trial assesses the impact of Daylight, a dCBT, among pregnant people with elevated anxiety symptoms who are minoritized based on race and ethnicity or sexual orientation and low income. This trial will also test whether there are salutary effects on symptoms often comorbid with anxiety in pregnancy, such as depression, stress, pregnancy-specific anxiety, and insomnia. This study is one of only 4 CBT trials to date [26,35] that examine effects on pregnancy-specific anxiety, a subtype of anxiety that remains understudied.

Given that all eligible participants in THRIVE will be part of California’s Medicaid insurance program, data collected will speak to the generalizability of dCBT for prenatal anxiety for low-income people and can be used to test whether race or ethnicity may impact intervention engagement or efficacy, moderation effects which are often confounded by differences in income across racial and ethnic groups. However, results will require replication among participants on Medicaid insurance from other geographic regions where enrollment is less robust and covers fewer health benefits [59].

### Conclusions

This dCBT adds to a very small literature on self-guided treatments for anxiety in pregnancy [60] and will provide valuable data surrounding adherence and engagement in the absence of regular clinical contact, which remains inaccessible for many low-income pregnant people.

## Appendix

Multimedia Appendix 1 SPIRIT (Standard Protocol Items: Recommendations for Interventional Trials) checklist.

Multimedia Appendix 2 Study team roles and responsibilities.

Multimedia Appendix 3 Consent form.

Multimedia Appendix 4 Adverse event criteria.

## Abbreviations

CBT: cognitive behavioral therapy
CTSI: Clinical & Translational Science Institute
dCBT: digital cognitive behavioral therapy
GAD-7: Generalized Anxiety Disorder- 7 Scale
HIPAA: Health Insurance Portability and Accountability Act
IRB: institutional review board
ISI-7: 7-item Insomnia Severity Index
LGBTQ+: lesbian, gay, bisexual, transgender, and queer
PHQ-9: Patient Health Questionnaire-9
PRAS-10: 10-item Pregnancy Related Anxiety Scale
PSI: Postpartum Support International
PSS-4: 4-item Perceived Stress Scale
RCT: randomized controlled trial
REDCap: Research Electronic Data Capture
SPIRIT: Standard Protocol Items: Recommendations for Interventional Trials
THRIVE: Transforming Health and Reducing Perinatal Anxiety through Virtual Engagement
UCSF: University of California, San Francisco
WGC: Western Copernicus Group
