# Supplementary material for: Transforming Health and Reducing Perinatal Anxiety Through Virtual Engagement: Protocol for a Randomized Controlled Trial
Source: JMIR Res Protoc. 2025 May 30;14:e70627. doi: 10.2196/70627 (PMC12166326; doi:10.2196/70627)
Supplement: Multimedia Appendix 2 [file resprot_v14i1e70627_app2.pdf]

Appendix B.

Roles and Responsibilities of Study Personnel and Funders

Contact PI/ MPI
Laura Jelliffe-Pawlowski, MS, PhD
Professor, Epidemiology & Biostatistics
Chair, Division of Lifecourse Epidemiology
Department of Epidemiology & Biostatistics
Institute of Global Health Sciences
University of California San Francisco School of Medicine
550 16th Street, 3rd Floor
San Francisco, CA 94158
Phone (HOPE Lab): 415-476-6132
Email: Laura.Jelliffe@ucsf.edu

Other MPIs (alphabetical)

Kacie Blackman, PhD
Assistant Professor, Department of Health Sciences
18111 Nordhoff Street
Northridge, CA 91330
Phone: 818-677-4918
Email: kacie.blackman@csun.edu

Bridgette Blebu, PhD, MPH
Investigator, Department of Obstetrics & Gynecology
The Lundquist Institute at Harbor-UCLA Medical Center
1124 West Carson Street
Torrance, CA 90502
Phone: 323-457-1909
Email: bridgette.blebu@lundquist.org

Jennifer Felder, PhD
Associate Professor of Psychiatry
Department of Psychiatry
University of California San Francisco School of Medicine
Box 1726, Floor 03, Room 336
San Francisco, CA 94158
Phone (Felder Lab): 415-476-7014
Email: Jennifer.Felder@ucsf.edu

Karen Tabb-Dina, PhD, MSW
Professor of Social Work
School of Social Work, Suite 2129

1010 W. Nevada | M/C 082
Urbana, IL 61801
Phone: 217-300-0200
Email: ktabb@illinois.edu

Roles and Responsibilities: The PIs have primary responsibility for the overall conduct of the study, including the safety of human subjects. The PIs will ensure appropriate (1) conduct of the informed consent process (e.g. that informed consent is obtained before proceeding with study procedures); (2) enrollment of study subjects; (3) collection and analysis of data; (4) implementation of study procedures to ensure consistent monitoring of subjects for possible adverse events; (5) review of adverse events and reporting to the WIRB if needed; and (6) maintenance of the privacy and confidentiality of study subjects. The PIs maintain ultimate responsibility for the project and for the safety of study participants. The PIs will be in contact with the research team on a regular basis to review the progress of the study and address any human subject issues that occur. These discussions may involve adverse event prevention measures, recruiting of appropriate study subjects, research staff training on protection of human subjects, as well as occurrence of adverse events, unexpected incidents, or protocol problems. PIs will also be involved in supervising study analyses and contribute to publications. This team of PIs will serve in lieu of a formal data monitoring committee, common in state funded grants.

Other Lead Study Staff

Scott Oltman, MS
Senior Epidemiologist
Department of Epidemiology & Biostatistics
Institute of Global Health Sciences
University of California San Francisco School of Medicine
550 16th Street, 3rd Floor,
San Francisco, CA 94158
Phone (HOPE Lab): 415-476-6132
Email: Scott.Oltman@ucsf.edu

Rebecca Baer, MPH
Senior Epidemiologist
Department of Obstetrics, Gynecology, Reproductive Sciences
University of California San Francisco School of Medicine
550 16th Street, 3rd Floor
San Francisco, CA 94158
Phone (HOPE Lab): 415-476-6132
Email: Rebecca.Baer@ucsf.edu

Carolyn Ponting, PhD
Postdoctoral Fellow
Department of Psychiatry

University of California San Francisco School of Medicine
Box 1726, Floor 03, Room 336
Phone (Felder Lab): 415-476-7014
Email: Carolyn.Ponting@ucsf.edu

Roles and Responsibilities: Study staff will be responsible for all enrollment procedures including reviewing study screening data, scheduling and conducting participant's study orientation sessions, and in the case of unblinded staff (i.e. Rebecca Baer, MPH) will randomize participants to Daylight or the waitlist control condition. Unblinded staff will also help participants troubleshoot any difficulties with the download or use of the Daylight app and will be responsible for administering $50 payments via Amazon gift card following surveys at enrollment, 3-, 6-, and 10- weeks post intervention start, and at 6 to 8 weeks postnatally. Staff will also be involved in data analysis and will contribute to study publications.

Other Clinical Partners

Larry Rand, MD
Professor of Obstetrics, Gynecology, Reproductive Sciences
Department of Obstetrics, Gynecology, Reproductive Sciences
University of California San Francisco School of Medicine (UCSF)
1825 4th Street, 3rd Floor
San Francisco, CA 94158
Phone: 415-885-7788
Larry.Rand@ucsf.edu

Elizabeth Rogers, MD
Professor of Pediatrics
Department of Pediatrics
University of California San Francisco School of Medicine (UCSF)
1855 4th Street, 2nd Floor, Room A-2432
San Francisco, CA 94158
Elizabeth.Rogers@ucsf.edu

Roles and Responsibilities: Dr. Rand and Dr. Rogers will be available as needed for consultation regarding recruitment, managing protocol deviations and data analysis and interpretation. Drs. Rand and Rogers will also serve as the study's medical monitors and will review any adverse medical events (Dr. Rand with respect to pregnant participants and Dr. Rogers with respect to infants) reported throughout the study. Drs. Rand and Rogers will advise on additional event assessment and any follow-up needed to determine the relationship between an adverse event and study participation.

Big Health
461 Bush Street #200
San Francisco, CA 94108
Contact:

Tali Ball, PhD (Senior Manager, Clinical Research)
Email: tali.ball@bighealth.com

Roles and Responsibilities: Dr. Ball will work with the institution of record (UCSF) to manage contracting so that the Daylight app is available for download for enrolled study participants. Dr. Ball will ensure that Big Health is able to provide data about app engagement and will help to connect the UCSF team with the appropriate contacts to troubleshoot any potential issues with app use. Big Health and affiliates will not have authority over data analyses or resulting publications.

Postpartum Support International
6706 SW 54th Ave
Portland, OR 97219
Contact:
Wendy Davis (Executive Director)
Phone: 503-277-3925
Email: wdavis@postpartum.net

The Preeclampsia Foundation
3840 West Eau Gallie Blvd Suite 104
Melbourne, FL 32934
Contacts:
Marne Silk (Research Manager)
Phone: 408-909-1589
Email: marne.silk@preeclampsia.org
Eleni Tsigas (Executive Director)
Phone: 321-421-6957
Email: eleni.tsigas@preeclampsia.org

Roles and Responsibilities: The Preeclampsia Foundation and Postpartum Support International will advertise the study on their respective platforms. These organizations may send IRB approved emails, post study flyers on their websites or suggest ways of increasing the reach of study information within California.
