# Supplementary material for: Transforming Health and Reducing Perinatal Anxiety Through Virtual Engagement: Protocol for a Randomized Controlled Trial
Source: JMIR Res Protoc. 2025 May 30;14:e70627. doi: 10.2196/70627 (PMC12166326; doi:10.2196/70627)
Supplement: Multimedia Appendix 3 [file resprot_v14i1e70627_app3.pdf]

IRB APPROVED
Jan 02, 2024

UNIVERSITY OF CALIFORNIA, SAN FRANCISCO
CONSENT TO PARTICIPATE IN A RESEARCH STUDY

TITLE: Transforming Health and Reducing PerInatal Anxiety through Virtual Engagement (the HOPE THRIVE STUDY)

Pregnant participant

PROTOCOL NO.: None
WCG IRB Protocol #20235218
23-40609

SPONSOR: The California Collaborative for Pandemic Recovery and Readiness Research (CPR3)

PRIMARY / CONTACT INVESTIGATOR: BLINDED

STUDY-RELATED PHONE NUMBER(S): 415.476.6132 (24 hours)

Taking part in this research is voluntary. You may decide not to participate, or you may leave the study at any time. Your decision will not result in any penalty or loss of benefits to which you are otherwise entitled.

If you have any questions, concerns, or complaints or think this research has hurt you, talk to the research team at the phone number(s) listed in this document.

This is a clinical research study. Research studies only include people who choose to take part. Take your time to make your decision about participating.

You are being asked to take part in this study because you are pregnant and meet other study eligibility criteria listed below including experiencing moderate to high levels of anxiety.

RESEARCH CONSENT SUMMARY

You are being asked for your consent to take part in a research study. This document provides a summary of this research. It describes the key information that we believe that most people need to decide whether to take part in this research. Later sections of this document will provide all relevant details.

What should I know about this research?

- This document outlines what is expected from you as part of this research

- If you don't understand, please ask questions by emailing us at: HOPE@ucsf.edu

Am I eligible to participate in this research?

Based on your screening survey you **are eligible to participate** in this study. Specifically, your screening survey results showed that:

- you are a California MediCal participant
- you are pregnant and 8-27 weeks gestation by ultrasound
- you are 18-years of age or older
- you are able to speak, read, and understand English
- you have a 9th grade level of education or more
- you have daily access to a web-enabled computer, smart phone, or tablet
- you are currently experiencing moderate to severe levels of anxiety (based on answers in the GAD-7 survey indicating a score of 10 or more)

Further, you were found as eligible for the study because:

- you have not received cognitive behavioral therapy (in person or digitally) for anxiety in the last 12-months
- you have not had a new or changed dose of prescription medication for anxiety, depressive symptoms, or poor sleep in the past 4 weeks
- you do not have a diagnosis of schizophrenia, psychosis, bipolar disorder, seizure disorder, substance use disorder, or for severe cognitive impairment (characterized by experiences like forgetting events, having decreased periods of alertness, decreased social awareness)
- you have not had any recent trauma to the head or brain damage
- you have no serious physical health concerns that have necessitated surgery or hospitalization in the last 6-months

☐ **If you do not think you meet/ still meet the above eligibility requirements, please check this box and do not continue with enrollment in the study.**

How many people will take part in this study?

Approximately 110 people will participate in this study.

How long will I be in this research?

You will participate in this study from the time you enroll until 6 to 8 weeks after your expected date of delivery.

Why is this research being done?

The purpose of this research study is to determine whether digital cognitive behavioral therapy (dCBT) for anxiety can help reduce anxiety-related symptoms in pregnant women and expectant mothers receiving MediCal insurance. Further, this research evaluates if use of dCBT for anxiety can help reduce levels of stress and depression or can help improve sleep in those who use it.

What happens to me if I agree to take part in this research?

- After confirming via completion of this consent form (completed and submitted online) that you would like to participate in this study you will:
    - Receive an enrollment survey by email which you will complete online. This survey asks about your background, pregnancy health, and about your general health and well-being. The survey will take about 10 to 20 minutes to complete.
    - In 1 to 7 days after completing your enrollment survey you will find out if you have been randomized to receive access to the investigational Daylight app immediately or in 10 weeks;
        - **You should only consent to participate in this study if you want to be part of it regardless of whether you are randomized to start using the Daylight app now or in 10-weeks;**
        - **You will receive compensation for participating in the study. This compensation is described in the "Will I be paid for taking part in this research?" section below. The compensation is the same regardless of what study group you are randomized to;**
        - If you were randomized to receive access to the Daylight app immediately, you will be sent instructions for downloading and starting to use the Daylight app in the same email;
        - If you were randomized to receive access to the Daylight app in 10-weeks, you will be notified that you will be sent instructions for downloading and starting to use the Daylight app in 10-weeks. This will be explained to you in the same email as report of your randomization.
    - If you were randomized to start using the Daylight app immediately, once you have downloaded the app and signed up to use Daylight, it is expected that you will use the app a minimum 1 time per week and ideally every few days;
        - Reminders to use the app will be sent to by app. You will also get a reminder from study staff once a week by text to use the app;
            - The Daylight app will guide you through four core program areas designed to help you address and manage anxiety. These program areas are called: "Tense and Relax", "Worry Time", "Thought

Challenger", and "Worry Exposure". Bonus tools and practice include: "Mindfulness", "Facing Avoidance", and "Problem Solving";
- Initial lessons/learning takes about 30 minutes;
- Then, ideally, you will spend 5-10 minutes every 1-2 days, for 6-weeks using the app with additional practice of techniques outside of app use;
- Weekly check-ins take place via the app with guided focus based on symptoms/improvement;
- Use of the Daylight app on at least a weekly basis (after you are given access and for 6-weeks afterwards) is required for continued study participation;
- The study requests that you use the app for 6-weeks. You are free to continue use after 6-weeks but this is not required for study participation.

- All participants, regardless of randomization grouping will receive surveys that take from 5 to 20 minutes to complete at 3-weeks, 6-weeks, and 10-weeks after the program start that ask about your health and well-being as well as about current experiencing of anxiety, stress, depression and sleep;
- All participants, regardless of randomization grouping will also receive a final survey at 6-8 weeks after expected date of delivery that will take about 20 to 25 minutes to complete that asks about your health and well-being, your labor and delivery experience, and the health of your baby. The survey also asks about your current experience of anxiety, stress, depression, and sleep;
- We ask that you try and answer all questions contained in the surveys as completely as possible. The more complete the data the more able we are to learn about factors affecting pregnancy and newborn health. Keeping this in mind, however, we understand if you feel the need to skip a question that feels unusually uncomfortable or stressful;
    - Questions related to anxiety, stress, depression, and sleep are required to be completed given their core importance to the study;
    - Several of the same questions are asked at different survey time points. This is because we are looking at how health and well-being may change over time and may be related to different timing in use of the DAYLIGHT app.
- If you were randomized to start using the app in 10-weeks, you will be sent information about downloading the app after completing your 10-week survey and will receive reminders for use in the same way as described above for those who start using the app immediately.

As part of your participation in the study, you can also choose to be considered for participation in an online focus group with 5 to 7 other study participants. This focus group will take place after completion of an initial 6-weeks of use of the Daylight app. The focus group will take approximately 60-90 minutes and will be done on a day and time that you express as being

convenient for you. Not all study participants will be invited to participate in a focus group discussion. You do not have to answer any questions that you do not want to. You will be asked to use a code name during the focus group in order to ensure that your privacy is protected.

- During the focus group discussion:
    - you will be asked to introduce yourself using your code name;
    - you will be asked to share your overall impression of the Daylight app and to talk about your experiences in using it and whether you would recommend it for use by others;
    - you will be asked whether you think use of the app helped reduce your feelings of anxiety or with other mental health challenges like with feelings of stress or depression or with sleep problems;
    - you will be asked whether you felt the app was culturally appropriate in terms of approach, content, voice, and avatar use;
    - you will be asked to share any other thoughts or input you have about using the app;
    - you will receive additional compensation for participation in this focus group (see description in the "Will I be paid for taking part in this research?" section below.

What side effects or risks can I expect from being in the study?

While this study involves no more than minimal risk, participants may experience some discomfort associated with this study due to the questions being asked around anxiety, stress, depression, and other factors. There may be other unknown risks. While the Daylight app is designed to help you address and manage anxiety and related symptoms, its use may also bring sensitive and distressing thoughts and memories to mind which could also be distressing to some. There is also some risk of your disclosing other personal information that makes you uncomfortable. ***Before enrolling in this study, it is important that you know that you do not have to share or discuss anything that you do not want to and that you are free to stop participating in the study or any related activities and dialogue at any time.***

Resources:

Pregnancy and new motherhood can be both a joyous and stressful time. Different people may need different kinds of help during this time. Throughout the course of the study we will share information with you about how to find resources in your community and get help if needed. We share it here as well so that you are aware of these resources.

If you need help finding information about local public health programs including about housing assistance, food programs, and other resources, please visit the HOPE connect website: https://hope.ucsf.edu/hope-connect. On this site you can also find mental health resources.

Information about perinatal anxiety can specifically be found on the Postpartum Support International Website here: https://www.postpartum.net/learn-more/anxiety/.

Free mental health resources are available for pregnant women and expectant mothers. The National Maternal Mental Health Hotline is a 24/7, free, confidential hotline for pregnant and new moms that is available in English and Spanish. You can call or text 1-833-TLC-MAMA (1-833-852-6262) to receive mental health help. TTY users can use a preferred relay service or dial 711 and then 1-833-852-6262.

If you are thinking about suicide, please call or text 988 or visit the 988 Suicide & Crisis Lifeline.

Are there any costs to me for taking part in this study?

No. The sponsor has agreed to pay for all items associated with this research study.

Will being in this research benefit me?

You may find that this study helps you feel better and that the Daylight app helps reduce your feelings of anxiety, stress, and depression or helps you experience better sleep. You may not experience such benefits. Even if you do not experience direct benefits from this study, your participation may benefit other pregnant women and expectant mothers by helping inform whether use of digital cognitive behavioral therapy helps improve mental health more generally in individuals who are pregnant.

What other choices do I have if I do not take part in this study?

The only alternative to participation in this study is not to participate.

How will information about me be kept confidential?

While researchers will do their best in making sure that all of the information you provide for this study is kept confidential, we cannot guarantee total privacy as your personal information may be given out if required by law.

If you agree to participate, no identifiers will be transcribed and stored with your surveys, on the Daylight app, or on any recording or transcripts related focus group participation. Each participant will be assigned a study ID that will be used on all documents. If information from this study is published or presented at scientific meetings, your name and other personal information will not be used.

Organizations that may look at and/or copy study records for research, quality assurance, and data analysis include:

- The Institutional Review Board (IRB) that reviewed this research (WCG IRB)
- Office for Human Research Protections (OHRP)
- Food and Drug Administration (FDA)
- Representatives of the University of California

While data collected as part of this research might be deidentified and used for future research or distributed to another investigator for future research without your consent, in no instance will any other investigator be given your name or contact information.

All data (recordings, transcripts, surveys, coded data) for the study is stored on encrypted servers kept behind UCSF firewalls where access is by remote access with dual authenticated password protection. No study data is kept or accessible on local devices. Answers to study questions and related analyses are kept separate from participant identifiers and contact information (name, phone, address, email) and are linked only by study ID number.

Throughout the study you will be asked to share sensitive personal information. All our tools are compliant with HIPAA, a law that protects the personal information of patients. However, we are mandated to report information if you report a risk of harming yourself or others, or if there is evidence of elder or child abuse.

If you report that you intend to harm yourself or others and you have consented for us to make contact if this happens, we will contact the personal contact/ support person you provided and your local emergency services. A space is provided at the bottom of this consent for designating a personal contact/ support person if you so choose.

Who can answer my questions about the study?

You can talk to the contact primary study investigator about any questions, concerns, or complaints you have about this study. Her direct email is: Laura.Jelliffe@UCSF.edu. You can also call her at: 415.476.6132. General study questions can be directed to study staff at: HOPE@ucsf.edu.

This research is being overseen by WCG IRB. An IRB is a group of people who perform independent review of research studies. You may talk to them at 855-818-2289 or clientcare@wcgclinical.com if:

- You have questions, concerns, or complaints that are not being answered by the research team.
- You are not getting answers from the research team.
- You cannot reach the research team.
- You want to talk to someone else about the research.
- You have questions about your rights as a research subject.

What happens if I am injured because I took part in this study?

It is important that you tell the contact primary study investigator if you feel that you have been injured because of taking part in this study. You can contact her at: Laura.Jelliffe@UCSF.edu. You can also call her at: 415.476.6132.

Treatment and Compensation for Injury:

If you are injured as a result of being in this study, the University of California will provide necessary medical treatment. The costs of the treatment may be billed to you or your insurer just like any other medical costs or covered by the University of California or the study sponsor depending on a number of factors. The University and the study sponsor do not normally provide any other form of compensation for injury. For further information about this, you may call the office of the UCSF Institutional Review Board at 415- 476-1814.

Can I stop being in the study?

You may withdraw from the study at any time for any reason.

Please let us know if you need to withdraw due to no longer being pregnant or for any other reason so that we may offer your spot to another interested person.

Study personnel retain the right to remove any participant from the study.

- Removal is automatic after 2 full weeks of non-use of the Daylight app (for those randomized to receive early treatment) and following 2 reminders sent by email and a text message.
- Removal is automatic after failure to complete any required survey and following 2 reminders by email and a text message.
- **Compensation does not continue after study withdrawal.**

Will I be paid for taking part in this research?

As part of the study, all participants will receive free, ongoing access to the Daylight app (during the study and until 12-months after the expected date of delivery for the current pregnancy).

You will receive up to $300 in Amazon gift cards as compensations for your participation in this study. Amazon gift cards are used for this purpose because they retain their full value and do not expire. A $50 electronic gift card will be sent to you by email within 1 to 7 days after: 1) completion and submission of your enrollment survey; 2) completion and submission of your 3-week survey; 3) completion and submission of your 6-week survey; 4) completion and submission of your 10-week survey; 5) completion and submission of your 6 to 8 week postnatal survey.

Individuals participating in an online focus group will receive an additional $50 amazon gift card delivered electronically by email within 1 to 7 days after participation.

Consent:

You have been given copies of this consent form and the Experimental Subject's Bill of Rights to keep.

PARTICIPATION IN RESEARCH IS VOLUNTARY. You have the right to decline to participate or to withdraw at any point in this study without penalty or loss of benefits to which you are otherwise entitled.

If you wish to participate in this study, you should sign below.

______________________	______________________	______________________
(**Printed** Name)	(**Signature**)	**(Date** mm/dd/yyyy**)**

Please also check one of the boxes below:

☐ **Please check this box if YOU WANT to be considered for participation in a focus group discussion with other participants after you complete your use of the Daylight app.** **Checking this box also expresses your understanding and consent to have the zoom focus group recorded and stored if you are selected for participation.*

☐ **Please check this box if YOU DO WANT to be considered for participation in a focus group discussion with other participants after you complete your use of the Daylight app.**

Information for an Optional Personal/ Support Contact

Your safety is of the utmost importance to us. If you would like to, please provide the name, email contact, and phone number of a Personal Support/ Contact Person. Please check in which instances you would like us to contact this person:

☐ **We lose contact with you and you are at-risk of being withdrawn from the study.** *In this instance, we will contact your personal support person by email.*

☐ **You report an intension to harm yourself or others.** *In this instance, we will contact your personal support person by phone and by text as well as local emergency services.*

<u>Optional Personal/ Support Contact Information</u>

______________________	______________________
(First Name)	(Last Name)
______________________	______________________
(email)	(mobile phone number)
______________________	
(optional other phone number)	

UNIVERSITY OF CALIFORNIA, SAN FRANCISCO

EXPERIMENTAL SUBJECT'S

BILL OF RIGHTS

The rights below are the rights of every person who is asked to be in a research study. As an experimental subject you have the following rights:

1) To be told what the study is trying to find out,
2) To be told what will happen to you and whether any of the procedures, drugs, or devices is different from what would be used in standard practice,
3) To be told about the frequent and/or important risks, side effects, or discomforts of the things that will happen to you for research purposes,
4) To be told if you can expect any benefit from participating, and, if so, what the benefit might be,
5) To be told of the other choices you have and how they may be better or worse than being in the study,
6) To be allowed to ask any questions concerning the study both before agreeing to be involved and during the course of the study,
7) To be told what sort of medical treatment is available if any complications arise,
8) To refuse to participate at all or to change my mind about participation after the study is started. This decision will not affect your right to receive the care you would receive if you were not in the study,
9) To receive a copy of the signed and dated consent form,
10) To be free of pressure when considering whether you wish to agree to be in the study.

Your study doctor will tell you if the research study is regulated by the Food and Drug Administration (FDA). If this study is FDA regulated, under the Federal Food, Drug, and Cosmetic Act (FD&C Act), FDA may inspect and copy all records relating to your participation in the study.

If you have other questions you should ask the researcher or the research assistant. In addition, you may contact the Institutional Review Board (IRB), which is concerned with protection of participant in research projects. You may reach the IRB office by calling: (415) 476-1814 from 8:00 AM to 5:00 PM, Monday to Friday, or by writing to the UCSF Human Research Protection Program, Box 1288, 490 Illinois Street, Floor 6, San Francisco, CA 94158, or by email at irb@ucsf.edu.

Call (415) 476-1814 or email irb@ucsf.edu for information on translations.
