# Supplementary material for: Transforming Health and Reducing Perinatal Anxiety Through Virtual Engagement: Protocol for a Randomized Controlled Trial
Source: JMIR Res Protoc. 2025 May 30;14:e70627. doi: 10.2196/70627 (PMC12166326; doi:10.2196/70627)
Supplement: Multimedia Appendix 4 [file resprot_v14i1e70627_app4.pdf]

Appendix D
THRIVE Study: Definition and Documentation of Adverse Events

Adverse events may be identified via self-report questionnaires or interviews or by other participant report. Upon identification of a possible adverse event, trained study staff will conduct an adverse event review to characterize the type and severity of the event and the relationship to the study intervention and study procedures. Dr. Felder will review psychiatric events and Drs. Rand and Rogers will review medical events (Dr. Rand with respect to pregnant participants and Dr. Rogers with respect to infants). These medical monitors will advise on additional event assessment and follow-up.

<u>Definition of adverse events</u>: Per the UCSF Human Research Protection Program, an adverse event is defined as any untoward or unfavorable medical occurrence in a human subject, including any abnormal sign (for example, abnormal physical exam or laboratory finding), symptom, or disease, temporally associated with the subject's participation in the research, whether or not considered related to the subject's participation in the research. For the purposes of this study, exacerbation in symptoms of primary or secondary focus in the study are not considered adverse events (anxiety, stress, depression, insomnia).

Per the UCSF Human Research Protection Program, a serious adverse event is defined as any adverse event that results in any of the following outcomes:

- Death
- Life-threatening adverse experience
- Inpatient hospitalization or prolongation of existing hospitalization
- Persistent or significant disability/incapacity
- Congenital anomaly/birth defect, or cancer
- Any other experience that suggests a significant hazard, contraindication, side effect or precaution that may require medical or surgical intervention to prevent one of the outcomes listed above
- Event that changes the risk/benefit ratio of the study

<u>Classification of adverse events:</u> The following guidelines will be used to describe severity.

- Mild: Participant is aware of symptoms, but is able to tolerate them, and no to minimal intervention is required.
- Moderate: Participant experiences enough symptoms to require intervention.
- Severe: Participant experiences symptoms or findings that require significant intervention or is life threatening. Severity is not synonymous with seriousness.

All adverse events (AEs) will have their relationship to study procedures, including the intervention, assessed by an appropriately-trained clinician based on temporal relationship and

his/her clinical judgment. The degree of certainty about causality will be graded using the categories below, per the UCSF Human Research Protection Program:

- Definitely related: An AE is definitely related to study participation if it is clear that the event was caused by study participation. A definitely related event has a strong temporal relationship, and an alternative cause is unlikely.
- Probably related: An AE is probably related when there is a reasonable possibility that the event is likely to have been caused by study participation. The AE has a timely relationship to the study procedure(s) and follows a known pattern of response, but a potential alternative cause may be present.
- Possibly related: An AE is possibly related when there is a reasonable possibility that the event might have been caused by study participation. A possibly related event may follow no known pattern of response and an alternative cause seems more likely. In other circumstances there may be significant uncertainty about the cause of the event, or a possible relationship to study participation cannot reasonably be ruled out.
- Unrelated: The cause of the AE is known and the event is in no way related to any aspect of study participation. If there is any uncertainty regarding AE causality, then the event must be assessed as possibly related to research participation and reported to the IRB as indicated. Often, the cause of an unrelated AE is disease progression.

<u>Expectedness:</u> Per the UCSF Human Research Protection Program, expected adverse events are defined as those that may be reasonably anticipated to occur as a result of the study procedures or study participation or is part of the normal disease process or progression.

An AE or suspected adverse reaction is considered "unexpected" if it is unlikely to occur in the study population, or it is unlikely to occur at the severity that has been observed. An unexpected AE also includes any AE that meets any of the following criteria:

- Results in subject withdrawal from study participation;
- Due to a deviation from the IRB approved study protocol.

The events below are considered expected and will not be reported to the IRB:

- COVID or other infection diagnosis
- Events that are expected during pregnancy, including but not limited to: depression, anxiety, worsening sleep quality, fatigue, gestational diabetes, hypertensive disorders, pregnancy loss including miscarriage and stillbirth, hyperemesis gravidarum, nausea or vomiting, increased susceptibility to infection (including urinary tract infection), vaginal bleeding, preterm labor, premature rupture of amniotic sac, preterm delivery, cesarean delivery, operative vaginal delivery, induction of labor.

- Events that are expected during the postpartum period, including but not limited to: lacerations and stitches, pain, postpartum hemorrhage, hypertension and hypertensive disorders, sleep disturbances, depression, anxiety.

Individual reports of AEs determined to be unrelated to research participation will not be reported to the IRB. Instead, these events will be documented, retained in the study files, and reported on an annual basis.

Time period and frequency: The occurrence of an adverse event (AE) or serious adverse event (SAE) may come to the attention of study personnel via participant report on the adverse event interview, other study questionnaires, or via other participant report.

All AEs will be captured on the adverse event report form. Information to be collected includes event description, time of onset, severity, relationship to study procedures, and time of resolution/stabilization of the event. All AEs will be documented appropriately regardless of relationship. All AEs will be followed to adequate resolution.

Any medical or psychiatric condition that is present at the time that the participant signs the study consent form will be considered as baseline and not reported as an AE. However, if the study participant's condition deteriorates at any time during the study, it will be recorded as an AE.

Changes in the severity of an AE will be documented to allow an assessment of the duration of the event at each level of severity to be performed. Documentation of onset and duration of each episode will be maintained for AEs characterized as intermittent.

The study team will record events with start dates occurring any time after the study consent is signed until the last day of study participation. Events will be followed for outcome information until resolution or stabilization.

Adverse event reporting: Adverse event reporting will be done in keeping with the WIRB/WCG Policy HRP-071. Specifically, adverse events will be reported to the WRB within 5 calendar days of PI awareness when the adverse event requires a change to the protocol or consent document. Specifically, this will include if there is:

- Unanticipated adverse device effect
- New or increased risk
- Protocol deviation that harmed a subject or placed subject at risk of harm
- Protocol deviation made without prior IRB approval to eliminate an immediate hazard to a subject
- Audit, inspection, or inquiry by a federal agency

- Written reports of federal agencies (e.g., FDA Form 483)
- Allegation of Noncompliance or Finding of Noncompliance
- Breach of confidentiality
- Unresolved subject complaint
- Suspension or premature termination by the sponsor, investigator, or institution
- Incarceration of a subject in a research study not approved to involve prisoners
- State medical board actions against physician researchers
